# Neighborhood social capital and infant physical abuse: a population-based study in Japan

**DOI:** 10.1186/s13033-016-0047-9

**Published:** 2016-02-27

**Authors:** Takeo Fujiwara, Yui Yamaoka, Ichiro Kawachi

**Affiliations:** Department of Social Medicine, National Research Institute for Child Health and Development, 2-10-1, Okura, Setagaya-ku, Tokyo, 158-8535 Japan; Department of Health Services Research, Faculty of Medicine, University of Tsukuba, Ibaraki, Japan; Department of Society and Behavioral Sciences, Harvard School of Public Health, Boston, MA USA

**Keywords:** Child abuse, Physical abuse, Shaken baby syndrome, Smothering, Spanking

## Abstract

**Purpose:**

We sought to investigate the relationship between neighborhood social capital and infant physical abuse using a population-based sample of women with 4-month-old infants in Japan.

**Methods:**

A questionnaire was administered to women who participated in a 4-month health checkup program (n = 1277; valid response rate, 80 %). We inquired about their perceptions of the level of trust in their neighborhood (an indicator of “social capital”) as well as the availability of support from their personal social networks. Infant physical abuse during the past month was assessed by self-reports of spanking, shaking or smothering.

**Results:**

The prevalence of infant physical abuse at 4 months of age was 9.0 % (95 % confidence interval [CI], 7.6–10.7 %). Women living in trusting neighborhoods were less likely to report infant physical abuse compared to those living in areas with low neighborhood trust (odds ratio [OR] 0.25, 95 % CI 0.06–0.97). In addition, women with supportive social networks were less likely to report infant physical abuse (OR 0.59, 95 % CI 0.36–0.99).

**Conclusions:**

In addition to one’s personal social network, social trust in the neighborhood was independently associated with lowered risk of infant physical abuse. To prevent infant abuse, interventions should consider strengthening community social bonds in addition to strengthening the social network of isolated mothers.

## Background

Child abuse is associated with a host of adverse outcomes including developmental delay [1, 2], poor academic performance [3, 4], mental disorders [5, 6], asthma [7, 8], obesity [9], cardiovascular disease [10], and even premature mortality later in adult life [11]. Moreover, the timing of child abuse is crucial [12–14]; the impact of child abuse is greater if abuse occurs at earlier developmental stages, such as infancy, due to the fragility of the infant brain as well as accumulation of damage over time [15–17].

The prevention of infant abuse depends on the identification of modifiable risk factors. Well-established risk factors for child abuse include young maternal age [18, 19], multiple births [20], low birth weight [21], maternal mental disorder, and parental history of childhood abuse [22]. In terms of the risk factors associated with the social environment, several studies have investigated the role of social support [23–28], and several studies examined the impact of neighborhood environment (i.e., contextual effect of neighborhood social environment on infant abuse) [29–35]. More precisely, the majority of the studies that have investigated the contextual effect of neighborhood social environment on child abuse focused on social disadvantages. For example, association with poverty or unemployment rate [30, 31], availability of child care or education resources [32], and even previous drug market activities [33], were used as objective measures of neighborhood social environment. In terms of the association with child abuse, perceived social disorder has been used as a subjective measure on neighborhood [34, 35]. The contextual effect of social disadvantage is important; however, social disadvantage is difficult to modify. Therefore, other social factors which are modifiable, such as social network or social capital within neighborhood [36], should instead be examined.

For the Project on Human Development in Chicago Neighborhoods, Molnar and colleagues examined the impact of neighborhood-level factors on the risk of parent-to-child physical aggression using three-level hierarchical linear models [37]. The authors found that a higher concentration of immigrants living in the neighborhood had significant protective effects on parent-to-child physical aggression after controlling child- and family-level factors, and the size of the social network (i.e., having a higher numbers of friends or relatives in the neighborhood) was significantly inversely associated with parent-to-child physical aggression, although only for Hispanic families. An important characteristic related to the concept of social networks is that of neighborhood social capital.

Social capital is defined as the resources acquired by individuals via their social networks in the community, school, work or other social settings [38]. A community with high social capital, for example, includes members who frequently assist each other and swap favors. A high degree of interpersonal trust in the social network is necessary for reciprocal cooperation to occur (i.e., a person trusts that the recipient of their favor will later reciprocate in kind in the future) [38]. Thus, social trust in the community is a core construct in neighborhood social capital. Previous studies reported a protective effect of social capital on child abuse and neglect [39–41]. Vinson et al. [40] reported that officially confirmed child abuse cases were spatially clustered, and that areas with high clustering were those in which residents reported a lack of attachment to their neighborhood, as well as few local friendships. Kim et al. [41] found that maternal community involvement and perceptions were inversely associated with their maltreatment behaviors, defined as psychological aggression, physical assault, and neglect. However, these studies did not examine physical abuse among infants using a population-based sample. Further, the impact of social capital (i.e., neighborhood environment) was not examined simultaneously with the impact of personal social networks. Social capital might have an independent effect regardless of the status of an individual’s social network.

Thus, the purpose of this study was to investigate the association between social capital and infant physical abuse using a population-based sample of women with 4-month-old infants in Japan.

## Method

### Sample

Details of this study have been published elsewhere [42]. In brief, we targeted all women (n = 1594) who were invited to participate in a 4-month health checkup program between June 2010 and January 2012 in Kamagaya City in Chiba Prefecture, Japan. Located near Tokyo, Kamagaya City’s population was 106,000 in 2010, and approximately 1000 births are recorded per year. A questionnaire was delivered to the target group via postal mail, and women handed in their completed surveys at each health checkup visit. In total, 1334 women responded to the questionnaire (response rate, 84 %). Our study was approved by the ethics committee of the National Institute for Public Health, Saitama, Japan. Ethics committee at National Center for Child Health and Development approved this study.

### Measure

#### Infant physical abuse

Infant physical abuse was measured as a maternal self-report of spanking, shaking, or smothering at least once during the past 1 month. Spanking was assessed by the question: “In the past month, how many times did you spank your baby when he/she was crying?” for which the responses ranged from “0 times,” “1 or 2 times,” “3–5 times,” “6–10 times,” and “11 or more times.” Shaking was assessed by the question: “In the past month, how many times did you violently shake your baby when he/she was crying?” for which the same response options were included. Smothering was assessed by the question: “In the past month, how many times have you smothered the mouth of your baby when crying, using your hands, a cushion, etc. during the past month?” with the same response categories. Mothers who responded with either spanking, shaking, or smothering their child one time or more were categorized as positive for infant physical abuse. As the questionnaire was anonymous, we could not refer positive response cases to child protection services.

#### Measurement of social capital

Social capital was assessed by perceived neighborhood trust and social support received from one’s personal social network. Perceived trust was assessed by the following question: “Do you think that people in your neighborhood trust each other?” with a 4-point Likert scale response, as follows: “Yes”, “Somewhat agree”, “Somewhat disagree”, and “No”. According to the responses, women were categorized as having high, middle-high, middle-low, or low neighborhood trust. Community-based social support was assessed by the following two questions: “Do you have someone to consult with in the community?” and “Do you have someone who can help you with child rearing in the community?” If women answered “yes” to one of these two questions, they were categorized as “having a supportive social network in the community”.

### Covariates

Questions on household characteristics (marital status, living together with grandparents or others, annual household income) and infant characteristics (sex, birth order, birth weight) were also included in the 4-month questionnaire. Annual household income was assessed by the following response categories: “≤2 million yen” (approximately USD 20,000), “2.1–4 million yen,” “4.1–6 million yen,” “6.1–8 million yen,” “8.1–10 million yen,” “10–15 million yen,” “15.1 million yen or more”, and “no answer”. Due to the distribution, the categories “8.1–10 million yen,” “10–15 million yen,” and “15.1 million yen or more” were collapsed for further analysis. The minimum/lowest income category, ≤2 million yen, was defined based on 50 % or lower of the median of average equivalent national household income [43].

### Analysis

We conducted a complete case analysis, i.e., complete responses to the questions about infant physical abuse and social capital indicators (n = 1277). The associations between infant physical abuse and social capital were analyzed using multiple logistic regression, adjusted for covariates (model 1), and simultaneously adjusted for neighborhood social capital indicators (model 2). All analyses were conducted using Stata/MP v12.0 software (StataCorp LP, College Station, TX, USA).

## Results

Table 1 shows the demographic characteristics of the study participants. The overall point prevalence of infant abuse was 9.0 % at 4 months. Most women were married (98.3 %), and not living with grandparents or relatives (89.2 %). For annual household income, 57.4 % of responders earned more than 4.1 million yen. Half of the infants were boys (50.4 %) and the firstborn child in the family (48.8 %). Low birth weight infants accounted for 9.2 % of the sample. Only three sets of twins (0.3 %) were identified in the sample.

**Table 1:** Characteristics of sample

	Total (n = 1277)		Abuse (n = 115, 9.0 %)		Non-abuse (n = 1162, 91 %)		P for Chi square
	N	%	N	%	N	%	
Household characteristics							
Marital status							
Married	1255	98.3	112	97.4	1143	98.4	0.503
Unmarried/divorced/other	19	1.5	3	2.6	16	1.4	
Missing	3	0.2	0	0.0	3	0.3	
Living with grand parents or others							
Yes	138	10.8	14	12.2	124	10.7	0.621
No	1139	89.2	101	87.8	1038	89.3	
Annual household income (million yen)							
≤2	33	2.6	5	4.4	28	2.4	0.474
2.1–4	369	28.9	34	29.6	335	28.8	
4.1–6	444	34.8	41	35.7	403	34.7	
6.1–8	197	15.4	13	11.3	184	15.8	
8+	92	7.2	6	5.2	86	7.4	
No answer	142	11.1	16	13.9	126	10.8	
Infant characteristics							
Sex							
Male	643	50.4	65	56.5	578	49.7	0.328
Female	630	49.3	50	43.5	580	49.9	
Missing	4	0.3	0	0.0	4	0.3	
Birth order							
First	623	48.8	61	53.0	562	48.4	0.558
Subsequent	651	51.0	54	47.0	597	51.4	
Missing	3	0.2	0	0.0	3	0.3	
Birth weight (g)							
<2500	118	9.2	15	13.0	103	8.9	
2500+	1136	89.0	99	86.1	1037	89.2	
Missing	23	1.8	1	0.9	22	1.9	

Table 2 describes the sample according to social capital indicators. Approximately 60 % of the participants perceived their level of neighborhood trust as middle-high or high. With regard to community-based social networks, the proportion of women with “someone to consult in the community” was 83.7 %, while the proportion of those with “someone to help with child rearing in the community” was 73.9 %; thus, 85.2 % of participants had access to either one or both types of supportive network.

**Table 2:** Characteristics of social capital and social network (N = 1277)

	N	%
Neighborhood trust		
Low	69	5.4
Middle-low	455	35.6
Middle-high	679	53.2
High	74	5.8
Social network		
Having someone to consult with in the community		
Yes	1069	83.7
No	207	16.2
Missing	1	0.1
Having someone who can help with child-rearing in the community		
Yes	944	73.9
No	330	25.8
Missing	3	0.2
Having a social network in the community		
Yes	1088	85.2
No	189	14.8

Table 3 presents the results of the logistic regression models. In the crude model, mothers who perceived higher neighborhood trust in their community were significantly less likely to abuse their infants. This association remained significant in model 1, which was adjusted for demographics, and in model 2, which was further adjusted for community-based social networks. Mothers who perceived high neighborhood trust were 75 % less likely to physically abuse their infant (OR 95 % CI 0.25, 0.06–0.97) compared to those who perceived low neighborhood trust in their community. Further, mothers who reported having supportive social networks in their community were significantly less likely to abuse their infant, even after adjusting for demographics and perceived neighborhood trust in model 2 (OR 0.59; 95 %CI 0.36–0.99).

**Table 3:** Odds ratio of social capital and social network for infant abuse (N = 1277)

	Infant abuse		Crude		Model 1		Model 2	
	N	%	OR	95 % CI	OR	95 % CI	OR	95 % CI
Social capital								
Low	12	17.4	Reference		Reference		Reference	
Middle-low	42	9.2	*0.48*	*0.24–0.97*	*0.49*	*0.24–0.99*	0.54	*0.26*–*1.11*
Middle-high	58	8.5	*0.44*	*0.23–0.87*	*0.46*	*0.23–0.92*	0.56	*0.27*–*1.15*
High	3	4.1	*0.20*	*0.05–0.75*	*0.20*	*0.05–0.76*	*0.25*	*0.06–0.97*
p for trend			*0.020*		*0.030*		0.127	
Social network								
No	27	14.3	Reference		Reference		Reference	
Yes	88	8.1	*0.53*	*0.33–0.84*	*0.53*	*0.33–0.86*	*0.59*	*0.36–0.99*

Italics signifies *p* < 0.05

*Model 1* adjusted for marital status, co-habitants, annual household income, infant’s sex, birth order, and low birth weight

*Model 2* model 1 plus social capital and social network

## Discussion

Our findings suggest that community social capital—as measured by perceptions of trust among neighbors, and the presence of supportive networks in the community—is protective of mother-to-infant physical aggression. Further, perceived neighborhood trust was protectively associated with infant physical abuse independent of the availability of supportive social networks in the community.

Our findings corroborate those of previous studies. Coohey [44] examined the relationship between different types of child maltreatment (physical abuse and neglect, only physical abuse, and only neglect), and womens’ social support in three components: structural properties, perceived support, and received support. The research targeted 150 maltreated women who attended a parenting class after child protection services became involved with their family and compared them with 150 mothers recruited via public schools in the community. The study found that mothers who both physically abused and neglected their children, and mothers who only neglected their children, were associated with having fewer members in their social networks and lower perceptions of support from their networks. Lower availability of emotional resources was associated with all three types of maltreatment. Williamson et al. [45] also reported that mothers reporting physical abuse or neglect of their child had lower levels of tangible social support and appraisal social support compared to mothers who did not maltreat their children. Neglectful parenting was found to have a significant inverse association with social capital, which was measured using neighborhood characteristics, willingness to take personal action, regular religious service attendance, and having a partner in the home [39].

Several mechanisms can be put forward as to why community social capital is protective of infant physical abuse [46]. First, psychosocial stress is lower among mothers living in a high social capital community. Strong bonds of mutual aid and reciprocity between neighbors can serve as a type of buffer in the event of crises and emergencies. Second, information about healthy child-rearing practices are more likely to diffuse quickly within cohesive networks, for example, how to appropriately deal with infants in distress, which is an important trigger for infant abuse. Thirdly, more cohesive communities may be better able to enforce healthy child-rearing norms, and they may be more effective in mobilizing appropriate resources, such as timely referral to social agencies when there is evidence of child abuse.

Our study has several limitations. First, we did not conduct a sociometric analysis of participants; our analyses are based on an assessment of ego-centered networks. To gain a better understanding of maternal social network resources, ideally we need to inquire about the amount and frequency of contact with network members, as well as the physical proximity of network members who could provide support during times of need. Secondly, we used only one item to assess perceived neighborhood trust. A more comprehensive approach to studying neighborhood social capital would assess other constructs such as collective efficacy and enforcement of norms, and examine the impact of social capital within a multi-level framework. Thirdly, experiences of infant physical abuse were self-reported and recalled from the time period of the infant’s birth until 4 months of age, and the severity of the outcomes were not assessed. Lastly, women who did not attend regular health check-ups were not evaluated in this study, thus our results might have underestimated the associations described.

In Japan, infants aged less than 1 year make up 42 % of fatalities caused by child maltreatment, and physical abuse is the leading cause of fatalities in this age group [47]. Our findings point to the potential promise of strengthening community bonds to prevent the occurrence of child abuse. One approach to strengthen community social capital is via home-visit programs conducted by health professionals or peer volunteers [48], especially targeting isolated mothers.

Alternatively, establishing community-based peer support groups for mothers with young children might be effective. In the elderly population in Japan, the establishment of community centers—known as “salons”—has been shown to be effective in strengthening community bonds [49]. Regular well-baby health checkups are a crucial window of opportunity for health professionals to assess women’s social networks and access to community-based support. This kind of assessment could be built into the collection of data that are already being gathered during antenatal visits to public health centers in Japan, which also provide screening for high-risk mothers.

Public health nurses can provide peer-support programs for mothers with few social networks. Dennis et al. [50] conducted a randomized controlled trial to evaluate the effect of peer support in the prevention of postpartum depression, and reported that telephone-based peer support might be effective in preventing postpartum depression. Further, they have shown that mothers endorsed emotional support (range 91.0–94.0 %), informational support (61.1–86.1 %), and appraisal support (48.0–92.5 %) [51]. In addition to home visits, peer support in the community may bolster social capital for isolated mothers.

Furthermore, neighborhood-based strategies to prevent child abuse and neglect, such as the “Strong Communities for Children” initiative in South Carolina in the United States [52, 53], might be effective. Based on its key message that a sense of collective responsibility among all people in the community can protect children [52], Strong Communities recruited volunteers and community organizations, and boosted various neighborhood activities to let residents “naturally” observe and respond to the needs of young families [54]. Through these activities, cases of child maltreatment and injuries indicative of maltreatment declined, positive parenting was observed, and low-resource communities experienced a greater level of mobilization, and enhanced reciprocal relationships between neighbors as well as a perception of household safety for neighborhood children [52]. These comprehensive community strategies should be considered, because communities with higher trust among neighbors may prevent infant physical abuse by mothers with limited social networks.

## Conclusion

In addition to one’s personal social network, social trust in the neighborhood was independently associated with lowered risk of infant physical abuse. To prevent infant abuse, interventions should consider strengthening community social bonds in addition to strengthening the social network of isolated mothers.

## Abbreviations

OR: odds ratio
CI: confidence interval
